# Efficacy of Oral Vitamin Supplementation in Inflammatory Rheumatic Disorders: A Systematic Review and Meta-Analysis of Randomized Controlled Trials

**DOI:** 10.3390/nu13010107

**Published:** 2020-12-30

**Authors:** Yann Nguyen, Johanna Sigaux, Jean-Guillaume Letarouilly, Pauline Sanchez, Sébastien Czernichow, René-Marc Flipo, Martin Soubrier, Luca Semerano, Raphaèle Seror, Jérémie Sellam, Claire Daïen

**Affiliations:** 1 Service de Médecine Interne, Assistance Publique-Hôpitaux de Paris (AP-HP), Nord, Hôpital Beaujon, Université de Paris, F-92100 Clichy, France; yann.nguyen2@aphp.fr; 2 Centre de Recherche en Epidémiologie et Santé des Populations, INSERM U1018, Université Paris-Saclay, F-94800 Villejuif, France; 3 Service de Rhumatologie, AP-HP, Hôpital Avicenne, F-93017 Bobigny, France; johanna.sigaux@aphp.fr (J.S.); luca.semerano@aphp.fr (L.S.); 4 INSERM U1125, Sorbonne Paris Cité, Université Paris 13, F-93017 Bobigny, France; 5 Service de Rhumatologie, Université de Lille, CHU Lille, F-59000 Lille, France; jeanguillaume.letarouilly@gmail.com (J.-G.L.); renemarc.flipo@chru-lille.fr (R.-M.F.); 6 Service de Rhumatologie, CHU de Montpellier, Université de Montpellier, F-34000 Montpellier, France; pauline.sanchez93@gmail.com; 7 Service de Nutrition, Centre Spécialisé Obésité, AP-HP, Hôpital Européen Georges Pompidou, Université de Paris, F-75015 Paris, France; sebastien.czernichow@aphp.fr; 8 Epidemiology and Biostatistics Sorbonne Paris City Center (CRESS), UMR1153, Institut National de la Santé et de la Recherche Médicale, F-75004 Paris, France; 9 Service de Rhumatologie, CHU Gabriel-Montpied, F-63000 Clermont-Ferrand, France; msoubrier@chu-clermontferrand.fr; 10 Service de Rhumatologie, AP-HP, Hôpitaux Universitaires Paris-Saclay—Hôpital Bicêtre, F-94270 Le Kremlin Bicêtre, France; raphaele.seror@aphp.fr; 11 INSERM U1184, Université Paris-Saclay, F-94270 Le Kremlin Bicêtre, France; 12 Service de Rhumatologie, Sorbonne Université, AP-HP, Hôpital Saint-Antoine, Inserm UMRS_938, FHU PaCeMM, F-75012 Paris, France; jeremie.sellam@aphp.fr

**Keywords:** rheumatoid arthritis, vitamin D, vitamin E, folic acid, vitamin K, meta-analysis, disease activity, diet

## Abstract

Background: We aimed to provide a systematic review and meta-analysis of randomized controlled trials assessing the effect of oral vitamin supplementation on symptoms and disease activity in patients with rheumatoid arthritis (RA), spondyloarthritis (SpA) and psoriatic arthritis (PsA). Methods: A systematic literature review and meta-analysis of randomized controlled trials including patients with inflammatory rheumatic diseases were performed using MEDLINE, EMBASE and abstracts from recent international rheumatology congresses. Studies were reviewed in accordance with PRISMA guidelines. We analysed clinical outcomes according to each type of vitamin supplementation. Results. The initial search yielded 606 articles. Of these, 13 studies were included in the qualitative synthesis: eight studied vitamin D supplementation, two assessed vitamin E supplementation, two folic acid, and one vitamin K, all of them on RA patients. No studies on SpA or PsA were selected. Oral vitamin supplementations were not associated with a reduction in RA activity (DAS-28 or pain) or RA flares. Conclusions: Despite their beneficial effects, the effects of vitamin supplementation on RA activity, if any, seem to be limited. Evidence on their efficacy on SpA or PsA activity is lacking. However, folic acid supplementation should be suggested to prevent methotrexate-related side effects, and vitamin D should be given to patients with vitamin D deficiency to prevent musculo-skeletal complications.

## 1. Introduction

Patients with inflammatory rheumatic diseases (IRD) are seeking natural and safe alternatives to complement their conventional anti-rheumatic therapies. Vitamins are over-the-counter supplements which might be attractive for patients as complementary medicines.

The family of vitamins is composed of various organic molecules with diverse chemical structure and biological function. Vitamin D was identified quite early as a putative candidate complementary treatment of rheumatoid arthritis (RA). Vitamin D is a hormone produced in the skin in the presence of UVB but can also be provided by dietary sources. Its immunomodulatory effects have been known for nearly three decades, and it has been shown that 1,25-dihydroxyvitamin D3 (or calcitriol, the biologically active metabolite of vitamin D) acts via the intracellular vitamin D receptor (VDR). VDR is ubiquitously present and specifically on the cells of the immune system [1]. Globally, 1,25-dihydroxyvitamin D3 inhibits pro-inflammatory Th1 and Th17 responses and promotes Th2 and T-reg responses, leading to regulation of the immune response of T effector cells [2,3,4], mechanisms that may be involved in the pathogenesis of RA. Moreover, 1,25-dihydroxyvitamin D3 prevented the development of arthritis and blocked its progression in a collagen-induced arthritis mouse model [5]. Oral vitamin D supplementation is available under vitamin D3 (cholecalciferol), or activated forms such as calcitriol, alphacalcidol or 22-oxa-1-alpha, 25-dihydroxy vitamin D3 (22-oxa-calcitriol), which has less hypercalcaemic activity than calcitriol.

Vitamin E is a major fat-soluble antioxidant present in plasma. As oxygen free radicals may play a role in the genesis and persistence of proliferative and destructive synovitis in RA [6], vitamin E might, therefore, restore a normal pool of reactive oxygen species scavengers and modulate eicosanoic acid production, via the action of tocopherol.

Vitamin K has been shown to inhibit the proliferation of fibroblast-like synoviocytes and the development of arthritis in mice [7] and is also important for bone metabolism [8].

Folic acid supplementation in IRD is widely used to reduce toxicity of methotrexate-related toxicity [9].

Hence, we aimed to provide a systematic literature review (SLR) and meta-analysis of randomized controlled trials assessing oral vitamin supplementation to reduce IRD symptoms and activity. This SLR was used to inform the recommendations of the French society of Rheumatology on diet in IRD.

## 2. Materials and Methods

This systematic review was conducted in accordance with the Preferred Reporting Items for Systematic Reviews and Meta-analyses (PRISMA) guidelines [10].

### 2.1. Search Strategy

We searched published studies indexed in MEDLINE and EMBASE databases from inception to June 2020 using the search strategy consistent with supplementation in different types of vitamins (A, B (including folic acid), C, D, E and K) and chronic IRD including RA, spondyloarthritis (SpA) and psoriatic arthritis (PsA), as described in Appendix A. A bibliography of selected retrieved articles was also manually searched and reviewed for inclusion. Conference abstracts from selected Rheumatology meetings (European League Against Rheumatism (EULAR) and the American College of Rheumatology (ACR)) and from Nutrition meetings (International Congress of Nutrition, European Nutrition Conference, American Society of Nutrition) from 2017 to 2019 were searched and manually reviewed for inclusion.

### 2.2. Inclusion Criteria

The studies fulfilling the following criteria were included into the systematic review: (1) Population: adults with confirmed IRD: RA, PsA or SpA. (2) Interventions: oral supplementations of vitamin A, B, C, D, E or K. (3) Comparators: placebo, standard of care or no intervention. (4) Outcome measures included clinical activity indexes (Disease Activity Score of 28 joints (DAS-28), Ritchie articular index, EULAR or ACR response for RA; Bath Ankylosing Spondylitis Disease Activity Index (BASDAI) for SpA; and psoriatic arthritis response criteria, EULAR or ACR response for PsA), Health Assessment Questionnaire Disability Index (HAQ), number of tender or swollen joints, visual analog scale for pain (VAS pain), morning stiffness duration, flares. (5) Types of studies: open-label or double blind randomized controlled studies. Subcutaneous or intravenous administrations of vitamins were excluded. Disagreement in the determination of the eligibility of each study was resolved by consensus.

### 2.3. Data Extraction

Data were independently collected using a standardized form by two authors (YN, CD). The data extraction form included study design, publication date, journal, location, disease, inclusion criteria, the type and length of the intervention, primary and secondary assessed outcome, number of participants, study population characteristic (including age, duration of the disease, clinical baseline characteristics and treatments), results in terms of disease activity, side effects and treatment adherence. If data were missing, authors of primary articles were contacted.

### 2.4. Quality Assessment

Risk of bias assessments were performed during data collection. The Cochrane Collaboration’s tool for assessing risk of bias [11,12] and the Jadad scale [13] were used to assess the risk of bias of each study. Records limited to abstracts were not assessed because of the paucity of available information.

### 2.5. Statistical Analysis

Studies assessing the same vitamin supplementation and the same outcome were considered for meta-analysis. The results were expressed as a mean difference for continuous variables. Significance was checked using a Z-test, and a *p*-value < 0.05 was considered as significant. Heterogeneity was assessed looking at confidence interval overlap between studies and using a Chi^2^ test. A I^2^ between 30 and 60% was interpreted as moderate heterogeneity and I^2^ > 60% as substantial heterogeneity. Random-effects models were used in case of apparent heterogeneity and fixed-model otherwise, to obtain adequate confidence intervals. If confidence intervals for the results of individual studies (generally depicted graphically using horizontal lines) have poor overlap, it generally indicates the presence of statistical heterogeneity. More formally, a statistical test for heterogeneity is available. This Chi^2^ (χ^2^, or chi-squared) test is included in the forest plots in Cochrane Reviews. It assesses whether observed differences in results are compatible with chance alone. A low *p*-value (or a large Chi^2^ statistic relative to its degree of freedom) provides evidence of heterogeneity of intervention effects (variation in effect estimates beyond chance).

A two-sided *p*-value < 0.05 was considered statistically significant. All analyses were conducted using Revman version 5.3.

## 3. Results

### 3.1. Study Selection

**Figure 1:**
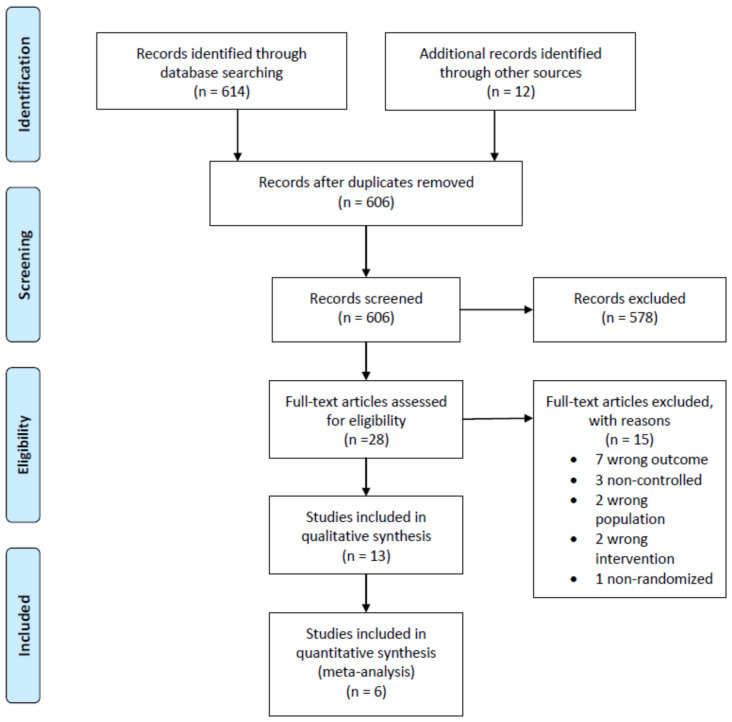
Preferred Reporting Items for Systematic Reviews and Meta-analyses (PRISMA) diagram.

The search process yielded 606 records (Figure 1). Of these, 28 were selected after review and assessed for eligibility. Of those, 13 studies were included in the qualitative synthesis. Among them, six studies (two on vitamin E and four on vitamin D) had sufficient data and were included in the meta-analysis. No unpublished relevant studies were obtained.

### 3.2. Study Characteristics

Baseline characteristics of the patients and inclusion criteria are summarized in Table 1. All studies were conducted on patients with RA: eight studied vitamin D supplementation, two assessed vitamin E supplementation, two folic acid and one vitamin K. No randomized controlled trials were found regarding PsA or SpA patients. All selected studies were published in English. Follow-up duration ranged from 3 weeks to one year.

Studies’ interventions and outcomes are reported in Table 2. Most studies assessed the efficacy on vitamin supplementation in reducing RA activity on patients with active RA, using clinical activity indexes (DAS-28, Ritchie articular index, EULAR or ACR response), HAQ, number of tender or swollen joints, VAS pain and morning stiffness duration. Only two studies assessed the efficacy of vitamin D supplementation on preventing the risk of RA flares in patients in remission.

The included studies involved 1218 RA patients. In one study [14], two groups assessing linoleic acid with or without vitamin E supplementation were not included in the review (only the placebo group and the vitamin E group were included). The main inclusion criteria were participants with RA diagnosed according to the 1987 ACR criteria, or 2010 ACR EULAR criteria. Most included patients had active RA except for two studies assessing vitamin D supplementation in RA flares, where participants were in remission [21,26]. 

### 3.3. Risk of Bias within Studies

**Figure 2:**
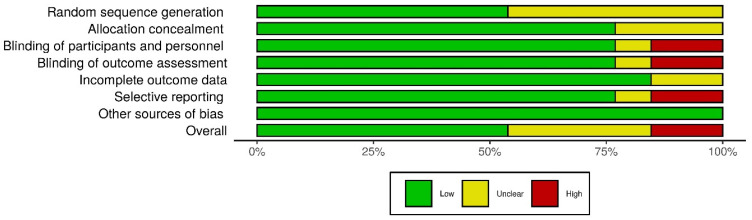
Distribution of risk-of-bias judgements within each bias domain of the Cochrane Collaboration’s tool.

**Figure 3:**
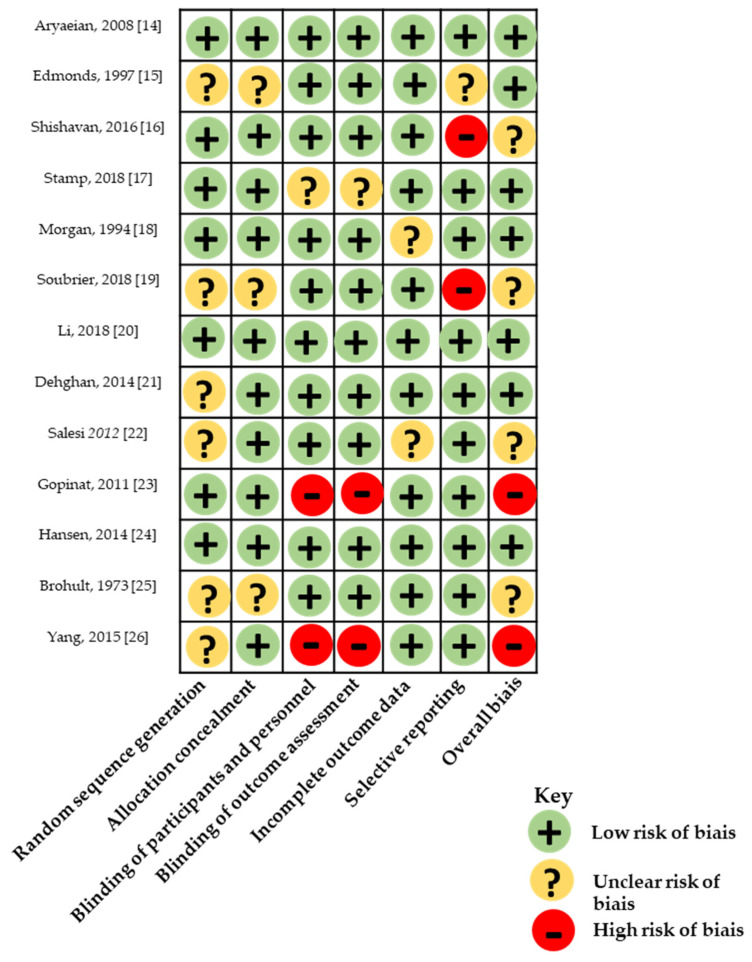
Quality assessment based on Cochrane collaboration tool.

Risk of bias in all 13 studies based on Jadad score of randomized controlled trials is reported in Table A1. All selected studies were randomized controlled trials, and only two of them were not double-blind and thus had a Jadad score of 3. Nine of the 13 studies had a Jadad score of 4 or more. Six studies did not report the used randomization sequence. Risk of bias was also assessed using Cochrane Collaboration’s tool (Figure 2 and Figure 3).

### 3.4. Outcomes

Outcomes of each study are summarized in Table 3 according to the type of vitamin supplementation.

#### 3.4.1. Vitamin E Supplementation

Two double-blind randomized controlled trials assessed the efficacy of a vitamin E supplementation (400 mg daily and 600 mg twice daily, respectively) on RA activity [14,15].

In their study, Aryaeian et al. aimed to assess the linoleic acid supplementation with or without vitamin E, hypothesizing a synergistic effect between those two molecules [14]. For the present review, only the vitamin E (without linolenic acid) and placebo group, including a total of 43 patients, were reported. There was no difference with placebo regarding DAS-28, VAS pain, SJC and TJC. 

In the second study including 42 patients, Edmonds et al. did not find any difference with placebo regarding Ritchie articular index, joint swelling or morning stiffness, but reported a significant decrease in VAS pain (−0.56 in the vitamin E group versus +0.54/10 in the placebo group, *p* = 0.006). However, such a decrease in VAS pain seemed to be small and not clinically pertinent. 

**Figure 4:**
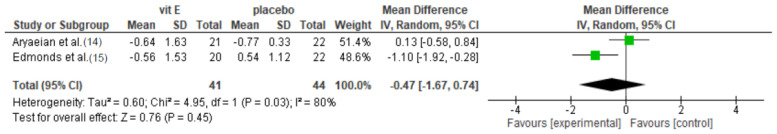
Meta-analysis of randomized controlled trials assessing the efficacy of vitamin E supplementation in visual analog scale for pain. Random effect model.

We performed a meta-analysis of both studies regarding the efficacy of vitamin E supplementation in VAS pain (Figure 4). On 85 patients in two groups, the mean difference [95% CI] in VAS pain was −0.47 [−1.67; 0.74] cm (*p* = 0.45), thus not statistically significant. There was a high heterogeneity in the studies (I² = 80%). 

Thus, the effect of vitamin E, if any, seems to be limited.

#### 3.4.2. Vitamin K Supplementation 

Only one study by Shishavan et al. conducted on 64 patients assessed the efficacy of vitamin K supplementation on RA. However, this study mainly focused on the biomarker of joint destruction (serum levels of matrix metalloproteinase-3) and reported that data on clinical activity (DAS-28) are limited. DAS-28 seemed to decrease in the vitamin K group (−12.56%, *p* = 0.041) compared with baseline. However, this decrease was not statistically significant compared with the placebo group. 

#### 3.4.3. Folic Acid Supplementation 

Two double-blind randomized controlled trials assessed the efficacy of folic acid supplementation in both reducing RA activity and preventing methotrexate-related adverse effects [17,18].

In their trial, Stamp et al. compared two doses of folic acid (5 and 0.8 mg/week) in patients with active RA (defined by a DAS-28 of 3.2 or greater) in 40 patients. There was no significant difference in the change in DAS-28 between the two regimens at 6 months: −0.13; 95% CI [−0.69; 0.43] in the 5 mg group versus −0.25; 95% CI [−0.87;0.37] in the 0.8 mg group; *p* = 0.78. There was also no significant difference in methotrexate-related adverse effects between the two groups. 

In the second trial, Morgan et al. aimed to determine the effect of two different weekly doses of folic acid (5 or 27.5 mg) compared with placebo on the efficacy and toxicity of methotrexate therapy on 94 patients. At either dose, folic acid supplementation did not affect the efficacy of methotrexate therapy as judged by joint indices for tenderness and swelling and HAQ. However, patients given folic acid supplementation had lower toxicity scores than participants given placebo (*p* < 0.001) with no difference between the two regimens. 

#### 3.4.4. Vitamin D Supplementation 

Eight studies assessed the efficacy of different vitamin D supplementation (calcitriol, cholecalciferol, alfacalcidol, 22-oxa-calcitriol) [19,20,21,22,23,24,25,26]. Among them, only three studies (Soubrier, Dehghan and Hansen) included patients with proven vitamin D deficiency. Data on the evolution on vitamin D levels under supplementation were available in these studies (Li, Salesi, Hansen).

Two of them (Dehghan and Yang) assessed the efficacy of vitamin D supplementation on the risk of flare on RA patients in remission [21,26]. Flares were defined by a DAS-28 score ≥ 3.2 by Yang et al., but not defined by Dehghan et al. None of the studies found a statistically significant reduction in RA recurrence with vitamin D supplementation. Risk of bias in Yang’s study was high due to the open-label design. Recurrence risk has already been meta-analysed, with an insignificant reduction in recurrence rates (risk difference −0.10, 95% CI [−0.21; 0.00]; *p* = 0.05), with a low heterogeneity (I² = 0%) [27].

**Figure 5:**
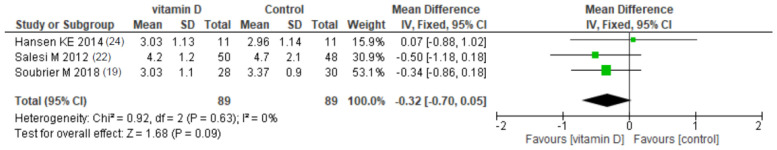
Meta-analysis of randomized controlled trials assessing the efficacy of vitamin D supplementation on DAS-28. Fixed effect model.

The other six studies assessed the efficacy of vitamin D supplementation on active RA patients, with or without vitamin D deficiency. Evolution of DAS-28 under vitamin D supplementation was assessed in three studies (Soubrier, Salesi and Hansen) [19,22,24]; none of them found a statistically significant change compared with placebo. We meta-analysed DAS-28 after the end of follow-up in intervention and control groups (Figure 5). Among 178 patients, the mean difference [95% CI] in DAS-28 was −0.32 [−0.70; 0.05] (*p* = 0.09), thus not statistically significant. There was no heterogeneity in the studies (I² = 0%).

**Figure 6:**
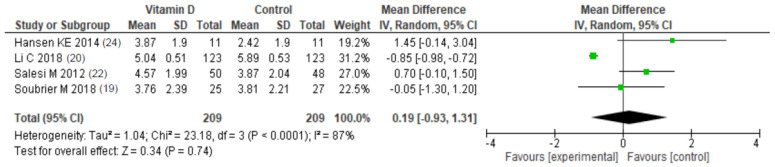
Meta-analysis of randomized controlled trials assessing the efficacy of vitamin D supplementation on visual analog scale for pain. Fixed effect model.

VAS pain reduction was assessed in five studies (Soubrier, Hansen, Salesi, Li and Gopinath) with contradictory results [19,20,22,24]. In their study, Hansen et al. found a statistically significant increase in VAS pain compared with placebo. Soubrier et al. and Salesi et al. did not find any differences between the vitamin D and placebo group. More recently, Li et al. found a statistically significant decrease in VAS pain in both 22-oxa-calcitriol and calcitriol groups compared with placebo, with no differences between those two groups. In their study, Gopinath et al. assessed the time to achieve pain relief and the proportion of patients with reduction in VAS pain. There were more patients with pain reduction in the intervention arm (*p* = 0.006). Risk of bias of this study was high due to the open-label design. We meta-analysed VAS pain after the end of follow-up in intervention and control groups (Figure 6). Among 420 patients, the mean difference [95% CI] in VAS pain was 0.24 [−0.88; 1.35] (*p* = 0.09), thus not statistically significant. There was high heterogeneity in the studies (I² = 88%).

In the study of Soubrier et al., after adjusting for age, gender, season, and initial vitamin D status, significant improvements in ESR and CRP levels were noted in the vitamin D group as compared to placebo groups, with *p*-values of 0.002 and 0.04, respectively. Risk of bias in Soubrier’s study was moderate. Finally, Brohult et al. assessed apparent objective and subjective symptom improvement and found a higher improvement rate in the calciferol group (67 vs. 36%). However, the absence of definition of the improvement makes those findings difficult to interpret.

## 4. Discussion

### 4.1. Summary of Evidence

There were only a few randomized controlled trials which investigated the efficacy of vitamin supplementation only on RA. Thus, there might be not enough evidence to properly determine the efficacy of vitamin supplementation on RA symptoms and recurrence.

Regarding vitamin E, only two studies evaluated the benefit of vitamin E supplementation with contradictory results. While there were no differences compared with placebo regarding SJC, TJC, DAS-28 in both studies, results were different for VAS pain, where Edmonds et al. found a benefit in vitamin E supplementation. The reduction in VAS pain was, however, not clinically relevant. Both studies were conducted at different time periods (1997 and 2008) with different treatments, and assessed two different regimens of vitamin E, which can partially explain those results. Nevertheless, pooling both studies in our meta-analysis led to no differences regarding VAS pain. Thus, the effect of vitamin E, if any, seems to be limited, and the evidence is too weak to recommend this supplementation for RA patients.

Regarding vitamin K supplementation, with only one study which did not find any statistically significant differences in DAS-28 reduction compared with placebo, the evidence is also too limited to recommend this supplementation.

Regarding folic acid supplementation, randomized controlled studies evaluated the efficacy in both reducing RA activity and preventing methotrexate-related adverse effects with different regimens. In those studies, there were no difference regarding DAS-28 reduction, joint indices for tenderness and swelling. However, patients with folic acid supplementation had lower methotrexate-related toxicity. Thus, while there might be no benefit of folic acid supplementation in RA patients not treated with methotrexate, folic acid should be given to patients treated with methotrexate to prevent its side effects, as stated in current treatment guidelines [9,28]. 

Finally, vitamin D supplementation has been assessed in two different situations: first, among RA patients in remission, for whom the risk of recurrence was evaluated in a previously published meta-analyses showing an insignificant reduction in recurrence rates; second, among active RA patients, to evaluate the benefit of this supplementation in reducing RA activity and symptoms. Regarding DAS-28 reduction, while none of the studies showed a benefit in vitamin D supplementation, the meta-analysis of the two studies (Hansen and Salesi) performed by Franco et al. [27] and ours assessing DAS-28 found no significant effect of vitamin D on DAS-28 reduction. Of note, both Franco’s and our meta-analyses were performed comparing the absolute values of DAS28 at follow-up between intervention and control group, rather than variations of DAS28 in the two groups, as these data were not available. As DAS-28 was relatively similar at baseline between the two groups, we believe that this method is acceptable. Regarding VAS pain, our meta-analysis did not show any significant effect of vitamin D. Nevertheless, studies assessing vitamin D supplementation are highly heterogeneous, and several factors could partly explain those discrepancies. Indeed, different vitamin D regimens were evaluated, and inclusion criteria varied across studies (patients in remission or active RA; vitamin D deficiency [19,21,24] or not), which makes comparisons across studies difficult. In addition, only three studies evaluated 25-OH vitamin D levels before and after supplementation (Table 3), and the increase in vitamin D levels was often limited, thus vitamin D supplementation might not be sufficient to provide any anti-inflammatory effect. Thus, if any, the effect of vitamin D on RA flares and RA activity seems to be limited. Evidence is currently lacking to recommend a systematic supplementation for this goal. However, as vitamin D deficiency is extremely frequent among the general population and RA patients, and is associated with an increased osteopenia and osteoporosis risk, especially within women and older patients with RA, patients with vitamin D deficiency should be supplemented for this specific aim [29,30]. Physicians may, however, be aware of potential side effects of vitamin D supplementation, including hypercalcemia and hypercalciuria [31]. 

### 4.2. Limitations

We acknowledge some limitations to our SLR and meta-analysis. First, there were numerous types of vitamin supplementation, even within the same category, different supplementation schemes, inclusion criteria and outcome definitions. Studies were conducted at different periods of time, with different standard of care regarding treatments, which can influence disease outcomes. 

Moreover, many studies were excluded from potential meta-analyses because important data could not be extracted such as the difference between baseline and end-of-treatment in both groups. Our meta-analyses on vitamin D pooled data at 3, 6 and 12 months which could be criticized. Finally, the quality of trials varied, as we had some concerns in randomization for six trials, and two trials (Gopinath and Yang) were not blinded, thus compromising the reliability of these data. 

## 5. Conclusions

Few studies assessed the benefit of vitamin supplementation on RA activity and recurrence rate. Evidence is currently lacking to recommend any vitamin supplementation to control disease activity, and further well-conducted RCTs are needed to complete our knowledge on this topic. Data regarding SpA or PsA are lacking. 

However, folic acid supplementation should be suggested to prevent methotrexate-related side effects, and vitamin D should be given to patients with vitamin D deficiency to prevent musculo-skeletal complications and in case of glucocorticoid treatment, as recommended in international guidelines for the prevention and treatment of glucocorticoid-induced osteoporosis [32,33]. 

## Figures and Tables

**Table 1:** Baseline characteristics of patients of the 13 studies included in the systematic review.

			Intervention Group		Control Group	
	Country		Age (Years)	Disease Duration (Years)	Age (Years)	Disease Duration (Years)
Aryaeian, 2008 [14]	Iran	ACR 1987, at least 2 years evolution	49.33	7.24	47.95	7.9
Edmonds, 1997 [15]	UK	ACR 1987, RAI ≥ 6 or MS ≥ 1 h	55.4	NR	52	NR
Shishavan, 2016 [16]	Iran	ACR 1987, 20–50 years old, DAS-28 < 5.1	38	3	39	7
Stamp, 2018 [17]	New Zealand	ACR 1987, under methotrexate and folic acid ≥ 3 months, DAS-28 ≥ 3.2	61.9	9.8	57.2	9.5
Morgan, 1994 [18]	UK	ARA 1987, 19–78 years, >6 months, active (TJC ≥ 6, SJC ≥ 3, MS ≥ 45 min, ESR ≥ 28 mm)	5 mg: 54 27.5 mg: 53.2	5 mg: 7.4 27.5 mg: 11.6	52.2	8.5
Soubrier, 2018 [19]	France	ACR 1987, DAS28 ≥ 2.6, vitamin D < 30 ng/mL	NR	NR	NR	NR
Li, 2018 [20]	China	ACR/EULAR criteria, TJC ≥ 4	22-Oxa: 48.5 Calcitriol: 49.6	22-Oxa: 6.5 Calcitriol: 6.6	51.1	6.9
Dehghan, 2014 [21]	Iran	ACR/EULAR criteria, in remission for > 2 years, vitamin D < 30 ng/mL	45	NR	4.7	NR
Salesi 2012 [22]	Iran	ACR 1987, DAS-28 ≥ 3.2	49.9	NR	50	NR
Gopinat, 2011 [23]	India	Early RA < 2 years without treatments	44.9	0.64	44.9	0.57
Hansen, 2014 [24]	USA	ACR 1987, vitamin D < 25 ng/mL	63	NR	63	NR
Brohult, 1973 [25]	Sweden	ACR 1987, >2 years evolution	53	NR	51	NR
Yang, 2015 [26]	China	ACR/EULAR criteria, in remission for >2 years	44.2	4.9	41.7	5.1

ACR: American College of Rheumatology; NR: not reported; RAI: Ritchie Articular Index; MS: morning stiffness; DAS-28: disease activity score—28 joints; TJC: tender joint count; SJC: swollen joint count; ESR: Erythrocyte Sedimentation Rate; EULAR: European League Against Rheumatism.

**Table 2:** Study characteristics of the 13 studies included in the systematic review sorted by vitamin type.

Study	Design	Population	Intervention		Controls		Outcome	Outcome Measurement
			Type	N	Type	N		
**Vitamin E**								
Aryaeian, 2008 [14]	Double blind RCT	102 randomized in 4 groups * incl. 51 in vitamin E or placebo groups → 43 completed	Vitamin E 400 mg/day for 12 weeks	21	Placebo for 12 weeks	22	DAS-28, VAS pain, SJC, TJC, Morning stiffness	12 weeks
Edmonds, 1997 [15]	Double blind RCT	42 randomized → 39 completed	Vitamin E 600 mg twice daily for 12 weeks	20	Placebo for 12 weeks	19	Ritchie articular index, morning stiffness, SJC, VAS pain	12 weeks
**Vitamin K**								
Shishavan, 2016 [16]	Double blind RCT	64 randomized → 58 completed	Vitamin K 10 mg/day for 8 weeks	30	Placebo for 8 weeks	28	DAS-28	8 weeks
**Folic acid**								
Stamp, 2018 [17]	Double blind RCT	40 randomized and completed	Folic acid 5 mg/day for 24 weeks	22	Folic acid 0.8 mg/day for 24 weeks	18	DAS-28	24 weeks
Morgan, 1994 [18]	Double blind RCT	94 randomized → 79 completed the study in three groups	Folic acid 5 mg/day or 27.5 mg/day for 1 year	25 + 26	Placebo	28	Ritchie articular index, Joint indices for tenderness and swelling, HAQ	1 year
**Vitamin D**								
Soubrier, 2018 [19]	Double blind RCT	59 randomized → 59 completed	Cholecalciferol 100,000 IU (frequency depending on the baseline vitamin D dosage) for 24 weeks	30	Placebo	29	HAQ, DAS-28, VAS pain	24 weeks
Li, 2018 [20]	Double blind RCT	369 randomized → 369 completed	22-Oxa-Calcitriol 50,000 IU/week for 6 weeks or Calcitriol 50,000 IU/week for 6 weeks	123 + 123	Placebo	123	SJC, VAS pain, HAQ	6 weeks
Dehghan, 2014 [21]	Double blind RCT	80 randomized → 80 completed	Cholecalciferol 50,000 IU/week for 6 months	40	Placebo	40	Number of flares	6 months
Salesi 2012 [22]	Double blind RCT	117 eligible → 98	Cholecalciferol 50,000 IU/week for 12 weeks	50	Placebo	48	DAS-28, TJC, SJC, VAS pain	12 weeks
Gopinat, 2011 [23]	Open label RCT	204 identified → 121 randomized → 110 completed	Calcitriol 500 IU + Calcium 1000 mg per day for 12 weeks	59	Calcium 1000 mg per day	62	Time to achieve pain relief, number of patients with VAS pain reduction	12 weeks
Hansen, 2014 [24]	Double blind RCT	711 contacted → 98 eligible → 22 randomized	Ergocalciferol 50,000 IU 3 times/week for one month then twice a month for 8 weeks	11	Placebo	11	HAQ, DAS-28, VAS pain	1 year
Brohult, 1973 [25]	Double blind RCT	49	Calciferol 100,000 IU per day for one year	24	Placebo	25	Objective and subjective symptom reduction	1 year
Yang, 2015 [26]	Open-label RCT	340 included→ 172 with vitamin D deficiency	Alfacalcidol 0.25 mcg twice a day for 24 weeks	84	Placebo	88	RA flare (DAS-28 > 3.2)	6 months

* Aryaeian et al. assessed four groups: linoleic acid, linoleic acid with vitamin E, vitamin E alone, and placebo. For this review, we only included the two following groups: vitamin E and placebo. RA: rheumatoid arthritis; RCT: randomized controlled trial; incl.: including; DAS-28: disease activity score—28; VAS pain: visual analog scale for pain; EULAR: European League Against Rheumatism; HAQ: Health Assessment Questionnaire; TJC: tender joint count; SJC: swollen joint count; IU: international units.

**Table 3:** Study results sorted by vitamin supplementation.

Study	Outcome	Intervention			Controls			*p*-Value (Intervention vs. Controls)
		Baseline	End of Treatment	Difference from Baseline	Baseline	End of Treatment	Difference from Baseline	
**Vitamin E**								
Aryaeian et al. [14]	DAS-28	4.59 (1.11)	NR	−0.77 (0.91) ^†^	4.35 (0.95)	NR	−0.31 (0.98)	>0.05
	VAS pain (cm)	4.02 (2.89)	NR	−0.64 (1.63)	5.11 (2.44)	NR	−0.77 (3.31)	>0.05
	SJC (n)	6.71 (9.67)	NR	−2.62 (9.94) ^†^	6.45 (8.89)	NR	−1.05 (7.74)	>0.05
	TJC (n)	3.76 (4.88)	NR	−1.29 (5.84) ^†^	2.86 (1.67)	NR	−0.68 (2.34)	>0.05
	Morning stiffness (hour)	1.09 (0.89)	NR	−0.66 (1.11) ^†^	1.27 (0.77)	NR	−0.05 (0.86)	>0.05
Edmonds et al. [15]	Ritchie’s index	15.9 (7.7)	15.3 (10.0)	NR	14.9 (8.8)	14.0 (12.1)	NR	>0.05
	Morning stiffness (min)	45	30	NR	30	20	NR	>0.05
	SJC (n)	9.2 (3.4)	9.9 (5.0)	NR	9.8 (5.4)	10.2 (5.6)	NR	>0.05
	VAS pain (cm)	4.63 (2.86)	NR	−0.56 (1.53)	3.74 (2.92)	NR	+0.54 (1.12)	0.006
**Vitamin K**								
Shishavan et al. [16]	DAS-28	NR	NR	−12.56% ^†^	NR	NR	NR	>0.05
**Folic acid**								
Stamp et al. [17]	DAS-28	3.5; range (2.4; 5.9)	NR	−0.13; 95% CI [−0.69; 0.43]	3.8; range [2.6; 5.8]	NR	−0.25; 95% CI [−0.87; 0.37]	0.78
Morgan et al. [18]	Joint indices for tenderness (n, min;max)	5 mg: 32 (6; 112) 27.5 mg: 34 (2;105)	5 mg: 21 (0; 90) ^†^ 27.5 mg: 14 (2; 41) ^†^	NR	34 (2; 99)	18 (4; 62) ^†^	NR	>0.05 >0.05
	Joint indices for swelling (n, min;max)	5 mg: 51 (14; 85) 27.5 mg: 43 (18;103)	5 mg: 14 (2; 41) ^†^ 27.5 mg: 13 (1; 58) ^†^	NR	45 (6; 85)	12 (0; 51) ^†^	NR	>0.05 >0.05
	HAQ (value, min;max)	5 mg: 2 (1; 3.8) 27.5 mg: 2 (1.1; 3.4)	5 mg: 1.2 (1; 2.8) ^†^ 27.5 mg: 1.2 (1; 2.6) ^†^	NR	1.8 (1; 3.4)	1.5 (1; 2.8) ^†^	NR	>0.05 >0.05
**Vitamin D**								
Soubrier et al. [19]	DAS-28	3.69 (0.96)	3.03 (1.1)	NR	3.76 (0.68)	3.37 (0.90)	NR	>0.05
	VAS pain (cm)	3.61 (1.64)	3.76 (2.39)	NR	3.76 (2.39)	3.81 (2.21)	NR	>0.05
	HAQ	NR	NR	−0.03 (0.23)	NR	NR	+0.08	0.11
	ESR (mm/h)	NR	NR	NR	NR	NR	NR	0.002 *
	C-reactive protein	NR	NR	NR	NR	NR	NR	0.04 *
Li et al. [20]	VAS pain (cm)	22-Oxa: 6.1 (0.59) Calcitriol: 5.8 (0.62)	22-Oxa: 5.2 (0.81) ^†^ Calcitriol: 5.04 (0.51) ^†^	NR	5.9 (0.52)	5.89 (0.53)	NR	22-Oxa: <0.05 Calcitriol: <0.05
	HAQ	22-Oxa: 1.33 (0.77) Calcitriol: 1.34 (0.79)	22-Oxa: 1.15 (0.1) ^†^ Calcitriol: 1.19 (0.28) ^†^	NR	1.31 (0.75)	1.29 (0.83)	NR	22-Oxa: <0.05 Calcitriol: >0.05
	Morning stiffness (mn)	22-Oxa: 146 (13) Calcitriol: 141 (12)	22-Oxa: 115 (15) ^†^ Calcitriol: 105 (14) ^†^	NR	135 (15)	130 (17) ^†^	NR	22-Oxa: <0.05 Calcitriol: <0.05
	Vitamin D (ng/mL)	22-Oxa: 15.72 (1.89) Calcitriol: 16.01 (1.98)	22-Oxa: 17.85 (1.09) Calcitriol: 17.92 (1.11)	NR	15.43 (1.53)	15.92 (4.56)	NR	22-Oxa: <0.05 Calcitriol: <0.05
Dehghan et al. [21]	Flares, n (%)	NA	7/40 (17.5%)	NA	NA	11/40 (27.5%)	NA	0.42
Salesi et al. [22]	DAS-28	5.4 (1.1)	4.2 (1.2)	NR	5.5 (1.3)	4.7 (2.1)	NR	>0.05
	VAS pain (cm)	6.26 (1.8)	4.57 (1.99)	NR	6.13 (2.18)	3.87 (2.04)	NR	>0.05
	TJC (n)	11.9 (5.8)	7.1 (5.1)	NR	12.8 (6.1)	9.2 (4.7)	NR	>0.05
	SJC (n)	2.7 (3.7)	1.1 (2.7)	NR	3.6 (4.4)	2.1 (3.2)	NR	>0.05
	Vitamin D (ng/mL)	42.8 (11.2)	50 (9.0)	NR	37.2 (13.2)	39.4 (12)	NR	<0.05
	ESR (mm/h)	35.8 (19)	26.2 (16.8)	NR	34.1 (18)	27.6 (17.3)	NR	>0.05
Gopinath et al. [23]	Time to achieve pain relief (days)	NA	21; range [7; 90]	NA	NA	21; range [7; 90]	NA	0.415
	% patients with reduction in VAS pain	NA	50; range [0; 100]	NA	NA	30; range [0; 30]	NA	0.006
Hansen et al. [24]	DAS-28	2.8; 95% CI [2.1; 3.3]	3.0; 95% CI [2.3; 3.8]	NR	2.7; 95% CI [2.1–3.3]	3.0; 95% CI [2.2; 3.7]	NR	0.96
	VAS pain (cm)	2.9; 95% CI [1.8; 3.7]	3.9; 95% CI [2.6; 5.2]	NR	2.9; 95% CI [1.8; 4.1]	2.4; 95% CI [1.1; 3.7]	NR	0.03
	HAQ	0.6; 95% CI [0.4; 0.9]	0.7; 95% CI [0.4; 1]	NR	0.6; 95% CI [0.4; 0.9]	0.4; 95% CI [0.2; 0.7]	NR	0.09
	Vitamin D (ng/mL)	25 (24)	30 (11)	NR	21 (9)	23 (11)	NR	<0.05
Brohult et al. [25]	Symptom reduction	NA	16/24 (67%)	NA	NA	8/25 (36%)	NA	0.01
Yang et al. [26]	Flares, n (%)	NA	16/84 (19%)	NA	NA	26/88 (29.5%)	NA	0.11

Results are presented as mean (SD) unless stated otherwise. Abbreviations: DAS-28: disease activity score—28; VAS pain: visual analog scale for pain; HAQ: Health Assessment Questionnaire; TJC: tender joint count; SJC: swollen joint count, mn: minutes; min: minimum; max: maximum; n: number; NA: not applicable; NR: not reported; ESR: erythrocyte sedimentation rate. ^†^ *p* < 0.05 between baseline and end of treatment. * after adjusting for age, gender, season and initial vitamin D status.

## Data Availability

Data are available upon reasonable request.

## Appendix A. Search Strategy

“Vitamins”[Mesh] OR vitamins OR vitamin OR « vitamin B » OR « vitamin C » OR “Vitamin D3 24-Hydroxylase”[Mesh] OR “Sodium-Coupled Vitamin C Transporters”[Mesh] OR “Vitamin D Response Element”[Mesh] OR “Vitamin B 6”[Mesh] OR “Vitamin E”[Mesh] OR “Vitamin D-Binding Protein”[Mesh] OR “Vitamin D”[Mesh] OR “Vitamin B 12”[Mesh] OR “Vitamin A”[Mesh] OR “Calcitriol”[Mesh] OR “Ascorbic Acid Deficiency”[Mesh] OR “aescorin” [Supplementary Concept] OR “Ergosterol”[Mesh] OR “Folic Acid”[Mesh] OR “vitamin D2 glucosiduronate” [Supplementary Concept] OR “vitamin D2 sulfate” [Supplementary Concept] OR “vitamin D3 glucosiduronate” [Supplementary Concept] OR “Tretinoin”[Mesh] OR “Cholecalciferol”[Mesh] OR “1,25-dihydroxy-26-(hydroxymethyl)vitamin D3” [Supplementary Concept] OR “alpha-lipoic acid, 4-aminobenzoic acid, aniline, benfotiamine, thioctic Acid, vitamin E drug combination” [Supplementary Concept] OR “1,25-dihydroxy-5,6-16-ene-vitamin D3” [Supplementary Concept] OR “1,25-dihydroxy-22-ene-vitamin D3” [Supplementary Concept] OR “1,25-dihydroxy-21-(3-hydroxy-3-methylbutyl)vitamin D(3)” [Supplementary Concept] OR “vitamin D 1-alpha hydroxylase” [Supplementary Concept] OR “dihydroxy-vitamin D3” [Supplementary Concept] OR “1,25-dihydroxy-16,23-diene vitamin D3” [Supplementary Concept] OR “Vitamin A”[Mesh] OR “Vitamin A” OR “Tretinoin”[Mesh] OR “Tretinoin” OR “Alitretinoin “[Mesh] OR « Alitretinoin » OR “Vitamin B Complex”[Mesh] OR « vitamin B » OR « vitamin B6 » OR « vitamin B12 » OR « Ascorbic Acid”[Mesh] OR « Ascorbic Acid” OR “Dehydroascorbic Acid” [Mesh] OR “Dehydroascorbic Acid” OR “Vitamin D”[Mesh] OR « Vitamin D » OR “Ergocalciferols”[Mesh] OR « Ergocalciferols » OR “Cholecalciferol”[Mesh] OR « Cholecalciferol » OR “Hydroxycholecalciferols”[Mesh] OR “Dihydroxycholecalciferols”[Mesh] OR “Calcitriol”[Mesh] OR “Vitamin E”[Mesh] OR « Vitamin E » OR “Tocopherols”[Mesh] OR « Tocopherols » OR “Tocotrienols”[Mesh] OR « tocotrienols ») AND (“Spondylitis, Ankylosing”[Mesh] OR ankylosis OR Spondylarthritis OR Spondylarthropathies OR “Arthritis, Rheumatoid”[Mesh] OR “rheumatoid arthritis” OR “rheumatoid” OR “Caplan Syndrome” OR “Felty Syndrome” OR “Rheumatoid Nodule” OR “Rheumatoid Vasculitis” OR “Arthritis, Psoriatic”[Mesh] OR “Psoriasis” OR “Arthritic Psoriasis” OR “Psoriatic Arthritis” OR “Psoriasis Arthropathica” OR “Psoriatic Arthropathy” OR “Arthropathies, Psoriatic” OR “Arthropathy, Psoriatic” OR “Psoriatic Arthropathies”) NOT “Administration, Cutaneous”[Mesh]) NOT “Administration, Topical”[Mesh] NOT “administration cutaneous” NOT “administration topical”.

## Appendix B

**Table A1:** Quality assessment based on Jadad score of randomized controlled trials reviewed.

Study	Randomization	Blinding	Account of All Patients	Total
Aryaeian et al. [14]	2	2	1	5
Edmonds et al. [15]	1	2	1	4
Shishavan et al. [16]	2	2	1	5
Stamp et al. [17]	2	2	1	5
Morgan et al. [18]	2	2	1	5
Soubrier et al. [19]	1	2	0	3
Li et al. [20]	2	2	1	5
Dehghan et al. [21]	1	2	1	4
Salesi et al. [22]	1	2	1	4
Gopinath et al. [23]	2	0	1	3
Hansen et al. [24]	2	2	0	4
Brohult et al. [25]	1	2	0	3
Yang et al. [26]	1	0	0	1
